# Nutraceutical Blends Promote Weight Loss, Inflammation Reduction, and Better Sleep: The Role of *Faecalibacterium prausnitzii* in Overweight Adults–A Double‐Blind Trial

**DOI:** 10.1002/mnfr.202400806

**Published:** 2025-02-21

**Authors:** Aline Boveto Santamarina, Victor Nehmi Filho, Jéssica Alves de Freitas, Lucas Augusto Moysés Franco, Roberta Cristina Martins, Joyce Vanessa Fonseca, José Antônio Orellana Turri, Mariana Tedesco Hufnagel, Daniel Pecoraro Demarque, Bruna Fernanda Rio Branco da Silva, Arianne Fagotti Gusmão, Eloísa Helena Ribeiro Olivieri, Erica de Souza, Esther Alves de Souza, José Pinhata Otoch, Ana Flávia Marçal Pessoa

**Affiliations:** ^1^ Laboratório de Produtos e Derivados Naturais Laboratório de Investigação Médica‐26 (LIM‐26) Departamento de Cirurgia Faculdade de Medicina da Universidade de São Paulo São Paulo Brazil; ^2^ Pesquisa e Desenvolvimento Efeom Nutrição S/A São Paulo Brazil; ^3^ Laboratório de Parasitologia Médica (LIM‐46) Departamento de Doenças Infecciosas e Parasitárias Faculdade de Medicina da Universidade de São Paulo São Paulo Brazil; ^4^ Laboratório de Investigação Médica em Protozoologia Bacteriologia e Resistência Antimicrobiana (LIM‐49) Departamento de Doenças Infecciosas e Parasitárias Faculdade de Medicina da Universidade de São Paulo São Paulo Brazil; ^5^ Departamento de Ginecologia e Obstetrícia Universidade de São Paulo Faculdade de Medicina São Paulo Brazil; ^6^ Laboratório de Farmacognosia Faculdade de Ciências Farmacêuticas Universidade de São Paulo São Paulo Brazil; ^7^ Laboratório Interdisciplinar em Fisiologia e Exercício Universidade Federal de São Paulo (UNIFESP) Santos Brazil; ^8^ International Research Center A.C. Camargo Cancer Center São Paulo Brazil; ^9^ Ambulatório Médico Monte Azul Associação Comunitária Monte Azul São Paulo Brazil; ^10^ Hospital Universitário da Universidade de São Paulo Faculdade de Medicina da Universidade de São Paulo São Paulo Brazil

**Keywords:** gut microbiota, inflammation, prebiotics, silymarin, sleep quality

## Abstract

This study explores the effects of a nutraceutical blend with prebiotics, β‐glucans, essential minerals, and silymarin on gut microbiota, inflammation, and sleep quality in obesity through microbiota reshaping and metabolic improvements over 90 days. A double‐blind, randomized trial was conducted on 77 participants divided into two groups receiving either a standard nutraceutical blend (NSupple) or a silymarin‐enriched blend (NSupple_*Silybum*). Fecal and plasma samples were collected at baseline and post‐supplementation for gut microbiota, metabolic, and inflammatory marker analysis. The results showed a reduction in body weight, waist‐to‐height ratio, total cholesterol, and fractions in the NSupple_*Silybum* group. There was a dysbiosis recovery shown by the increase in beneficial gut bacteria, such as Lentisphaerae phylum, *Lactobacillus* and *Faecalibacterium* genera, and *Faecalibacterium prausnitzii* in the NSupple group, with a concurrent reduction in *Adlercreutzia* and *Sutterella* in the NSupple_*Silybum* group. Both groups demonstrated improved inflammatory profiles by the reduced TNF‐α/IL‐10 ratio, reduced cortisol levels, and reduced Firmicutes/Bacteroides ratio. Additionally, improvements in sleep quality were associated with reductions in pro‐inflammatory cytokines and improved microbiota composition. The nutraceutical blend reshaped gut microbiota, enhanced anti‐inflammatory species, and improved metabolic and sleep parameters, highlighting its potential as a nutritional strategy for managing obesity and reducing inflammation.

## Graphical abstract

This study explored how a 90‐day nutraceutical blend with prebiotics, minerals, and silymarin affects the gut‐brain axis in overweight adults. Results showed improved gut microbiota, reduced inflammation, and better sleep, mood, and quality of life, suggesting a strong link between gut health and mental well‐being. Assessments were done at the start and end of the study.

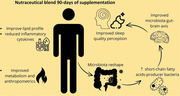

**Abbreviations**
ALT: alanine transaminase
AST: aspartate transaminase
AST/ALT: ratio aspartate transaminase/alanine transaminase ratio
BMI: body mass index
BRUMS: Brunel Mood Scale
BSFS: Bristol Stool Form Scale
CBA: cytometric bead array
CCL5/RANTES: C‐C motif ligand 5/regulated‐on‐activation‐normal‐T‐cell‐expressed‐and‐secreted
CONSORT: Consolidated Standards of Reporting Trials
C‐RP: C‐reactive protein
CXCL10: chemokine interferon‐γ inducible protein 10
CXCL9 MIG: monokine induced by interferon‐gamma
ESS: Epworth Sleepiness Scale
FOS: fructooligosaccharides
Gamma‐GT: gamma‐glutamyl transferase
GOS: galactooligosaccharides
HDL‐c: high‐density lipoprotein‐cholesterol
HOMA‐IR: Homeostatic Model Assessment for Insulin Resistance
HPA: hypothalamic‐pituitary‐adrenal axis
IgA: Immunoglobulin A
IgG: Immunoglobulin G
IL‐10: interleukin‐10
IL‐12p70: interleukin‐12p70
IL‐1β: interleukin‐1 beta
IL‐6: interleukin‐6
IL‐6/IL‐10 ratio: interleukin‐6/interleukin‐10 ratio
IL‐8: interleukin‐8
LDL‐c: low‐density lipoprotein‐cholesterol
MSQ‐BR: Brazilian Portuguese Version of the Mini‐Sleep Questionnaire
non‐HDL‐c: non‐high‐density lipoprotein‐cholesterol
OTUs: operational taxonomic units
PCoA: principal coordinate analysis plot
PSQI: Pittsburgh Sleep Quality Index
REM: rapid eye movement
SCFAs: short‐chain fatty acids
SNS: sympathetic nervous system
SWS: slow‐wave sleep
TNF‐α: tumor necrosis factor‐alpha
TNF‐α/IL‐10 ratio: tumor necrosis factor‐alpha/interleukin‐10 ratio
TSH: thyroid‐stimulating hormone
VLDL‐c: very low‐density lipoprotein‐cholesterol
WC‐IC: waist circumference at the iliac crest
WC‐mid: waist circumference at the mid‐abdomen
WHOQoL‐BREF: World Health Organization Quality of Life Instrument‐Short Form
WHR: waist‐to‐hip ratio
WHtR: waist‐to‐height ratio

## 1 Introduction

The human gut microbiota, a complex community of trillions of microorganisms, plays an essential role in maintaining overall health [1]. This intricate ecosystem is involved in numerous physiological processes, including digestion, immune modulation, and the synthesis of essential vitamins and bioactive compounds [2]. The composition and diversity of gut microbiota are crucial in preventing and managing various metabolic diseases, particularly obesity. In obesity, dysbiosis—a state of microbial imbalance—often occurs, characterized by a reduction in microbial diversity and an increase in harmful bacteria [3]. This imbalance can disrupt the gut barrier, leading to increased intestinal permeability, and triggering systemic inflammation [4]. The management of gut microbiota composition is, therefore, a critical aspect of preventing and treating obesity and its associated metabolic disorders.

Nutraceutical blends, particularly those containing prebiotics, have gained attention for their ability to modulate gut microbiota [5]. Prebiotics like fructooligosaccharides (FOS) and galactooligosaccharides (GOS) are non‐digestible components that selectively stimulate the growth and activity of beneficial bacteria, such as *Faecalibacterium* and *Lactobacillus*, in the gut. These bacteria are known for their ability to produce short‐chain fatty acids (SCFAs), such as butyrate, which play a crucial role in maintaining gut health by strengthening the gut barrier, reducing inflammation, and modulating the immune response and neurotransmitter metabolism [6]. Beta‐glucan is a potent nutraceutical component with immunomodulatory properties that can enhance the innate immune response by activating macrophages and natural killer cells, thereby helping to maintain a balanced immune environment within the gut [7]. Zinc, selenium, and magnesium are also essential for immune function, reducing oxidative stress, and metabolic health. Zinc aids immunity and cell division, selenium acts as an antioxidant and supports thyroid function, and magnesium is a key factor in energy production [8, 9, 10]. Together, these prebiotics and immunomodulatory agents can synergistically promote the growth of beneficial gut microbiota and enhance the gut's immune functions as tested by our group in preclinical studies [11, 12, 13, 14]. Their inclusion in a nutraceutical blend can help improve immune and metabolic health, especially in those with obesity and metabolic diseases.

Inflammatory cytokines and cortisol levels are closely linked to gut microbiota composition, particularly in the context of obesity and metabolic diseases [15]. Dysbiosis can lead to the overproduction of pro‐inflammatory cytokines, contributing to chronic low‐grade inflammation—a hallmark of obesity and metabolic syndrome [16]. This inflammation is further exacerbated by elevated cortisol levels, a stress hormone that, when chronically high, can lead to insulin resistance, fat accumulation, and disruptions in sleep and mood [17]. The overproduction of pro‐inflammatory cytokines has been shown to interfere with sleep architecture, particularly by reducing slow‐wave sleep (SWS) and altering rapid eye movement (REM) sleep [18]. This creates a vicious cycle where poor sleep exacerbates inflammation, and heightened inflammation further disrupts sleep [19]. The modulation of gut microbiota through a targeted nutraceutical blend could potentially reduce the production of these pro‐inflammatory cytokines and normalize cortisol levels, thereby improving metabolic health as previous results suggest [20, 21, 22]. Such modulation could also enhance the gut‐brain axis, a bidirectional communication network that connects the gut microbiota with the central nervous system, immune signaling, and the production of microbial metabolites like SCFAs [23], leading to improvements in sleep quality and reductions in mood disorders, which are often comorbid with obesity.

Previous studies have highlighted the efficacy of nutraceuticals in addressing the interconnected challenges of obesity, inflammation, and sleep disturbances [24]. By targeting the gut microbiota, nutraceuticals promote microbial shifts that enhance metabolic flexibility and reduce systemic inflammation [25]. Components like prebiotics, β‐glucans, and polyphenols have demonstrated their ability to increase beneficial taxa, such as *Faecalibacterium prausnitzii* and *Lactobacillus*, which produce SCFAs. These metabolites strengthen gut barrier integrity and modulate immune responses, contributing to improved metabolic markers, including reductions in body weight and insulin resistance [26]. Additionally, SCFA, as well as sleep‐enhancing compounds, including polyphenols and minerals like magnesium, regulate the hypothalamic‐pituitary‐adrenal (HPA) axis and reduce pro‐inflammatory cytokines that disrupt sleep architecture [27, 28].

The combination of compounds in the present study leverages their synergistic potential to modulate gut microbiota and mitigate inflammation, addressing key aspects of metabolic syndrome. Silymarin, a potent antioxidant and anti‐inflammatory polyphenolic compound, improves liver health [29]. Simultaneously, prebiotics such as FOS and GOS selectively enhance the growth of SCFA‐producing bacteria, reducing systemic inflammation and promoting immune balance [30]. This integrative approach highlights the promise of nutraceutical strategies in combating the systemic inflammation and dysbiosis associated with metabolic disorders.

Given the interconnectedness of gut microbiota, inflammation, sleep, and mood in the context of obesity and metabolic diseases, a nutraceutical blend that combines prebiotics, essential minerals, and immunomodulatory agents holds promise as a synergistic intervention [11, 12, 20, 22]. By reshaping the gut microbiota, this blend could reduce systemic inflammation, lower cortisol levels, and improve sleep and mood, ultimately enhancing the quality of life for people with obesity and metabolic diseases. The inclusion of silymarin, a polyphenolic compound known for its antioxidant and anti‐inflammatory properties, further strengthens the blend's potential efficacy. Silymarin can protect against oxidative stress and liver damage and act as a flavonobiotic compound reshaping gut microbiota, thus providing additional benefits in this population [31].

This comprehensive nutraceutical approach addresses multiple facets of health impacted by obesity and metabolic diseases, offering a potential avenue for improved health outcomes through targeted nutritional intervention. Therefore, the present study aims to elucidate the effects of a nutraceutical blend on gut microbiota reshaping and its subsequent impact on body composition, inflammation, and sleep quality. These benefits are attributed solely to the supplementation of a synergistic nutraceutical blend that includes prebiotics FOS and GOS, essential minerals, the immunomodulatory molecule beta‐glucan, and silymarin polyphenols.

## 2 Experimental Section

### 2.1 Ethics Committee Approval

This research complied with the Declaration of Helsinki [32] and received formal approval from the Ethics Committee for the Analysis of Research Projects (CAPPesq) of the HC‐FMUSP Research Ethics Committee under CAAE number 39984320.5.0000.0068. Additionally, it was approved by the Brazilian National System of Genetic Registration (SisGen) under registration number AC29D69 and registered on ClinicalTrials.gov (NCT04810572) 2 on March 23, 2021.

### 2.2 Participant Recruitment

From January 3, 2021, to September 6, 2021, volunteer residents from the southeastern region of Brazil were recruited for this clinical trial through the outpatient clinic *“Ambulatório Monte Azul”* (São Paulo, Brazil) and online advertisements. Data collection concluded on December 20, 2021. This double‐blind, randomized trial assessed participants at baseline (T0) and after 90 days of supplementation (T90). Inclusion and exclusion criteria and supplementation protocols followed the guidelines established by Nehmi‐Filho et al. [21]. Exclusion criteria included the use of insulin injections, corticosteroids, or non‐steroidal anti‐inflammatory drugs for more than 15 days; diagnosis of AIDS or hepatitis; pregnancy; and ongoing chemotherapy treatment.

### 2.3 Dosage Regimen and Formulas Composition

Two different formulations were tested herein as previously published by Nehmi‐Filho et al. [21], namely, NSupple and NSupple_*Silybum*. The formulations’ composition and dosage are described in Table 1. The precise amount of each active component was not disclosed due to the patent register requirements (patent number: BR 10 2020 016156 3), which can be accessed upon request. The active compounds dosages applied were determined in accordance with the EFSA [33], RDA [34], and FDA [35] guidelines as non‐pharmacological. The pharmaceutical team at *“Solis Magistral Farmácia Homeopatia Sensitiva”* (São Paulo, Brazil) was responsible for keeping the double‐blinding point of this study.

**TABLE 1:** Supplements formulation description.

Capsules components^*^	NSupple	NSupple_ *Silybum*
Zinc (Zn)^a^	1%	1%
Magnesium (Mg)^a^	1%	1%
Fructooligosaccharide (FOS)^b^	45%	45%
Selenomethionine (Se)c	0.01%	0.01%
Galactooligosaccharide (GOS)^c^	10%	10%
1.3/1.6‐(β‐glycosidic bonds) yeast β‐glucans (*Saccharomyces cerevisiae*)^c^	6%	6%
Tixosil^c^	5%	5%
Silymarin seed extract (*Silybum marianum* (L.) Gaertn.)^d^	—	3.11%

* four capsules of dosage.

^a^ Purifarma Distribuidora Química e Farmacêutica, São Paulo, Brazil.

^b^ NutraFlora, Westchester, Illinois, USA.

^c^ Biorigin, São Paulo, Brazil.

^d^ SM Empreendimento Farmacêutica LTDA, São Paulo, Brazil.

Volunteers were advised to take two supplement capsules in the morning and two capsules in the evening. The daily dosage of the supplement remains within a non‐pharmacological range that could theoretically be achieved through diet, classifying it as a nutritional supplement. However, the dietary sources of the prebiotic components in these formulations are typically not easily accessible in a regular diet. Therefore, supplementation offers a practical tool to ensure consistent intake of these health‐promoting nutrients.

### 2.4 Randomization and Study Progression

**FIGURE 1:**
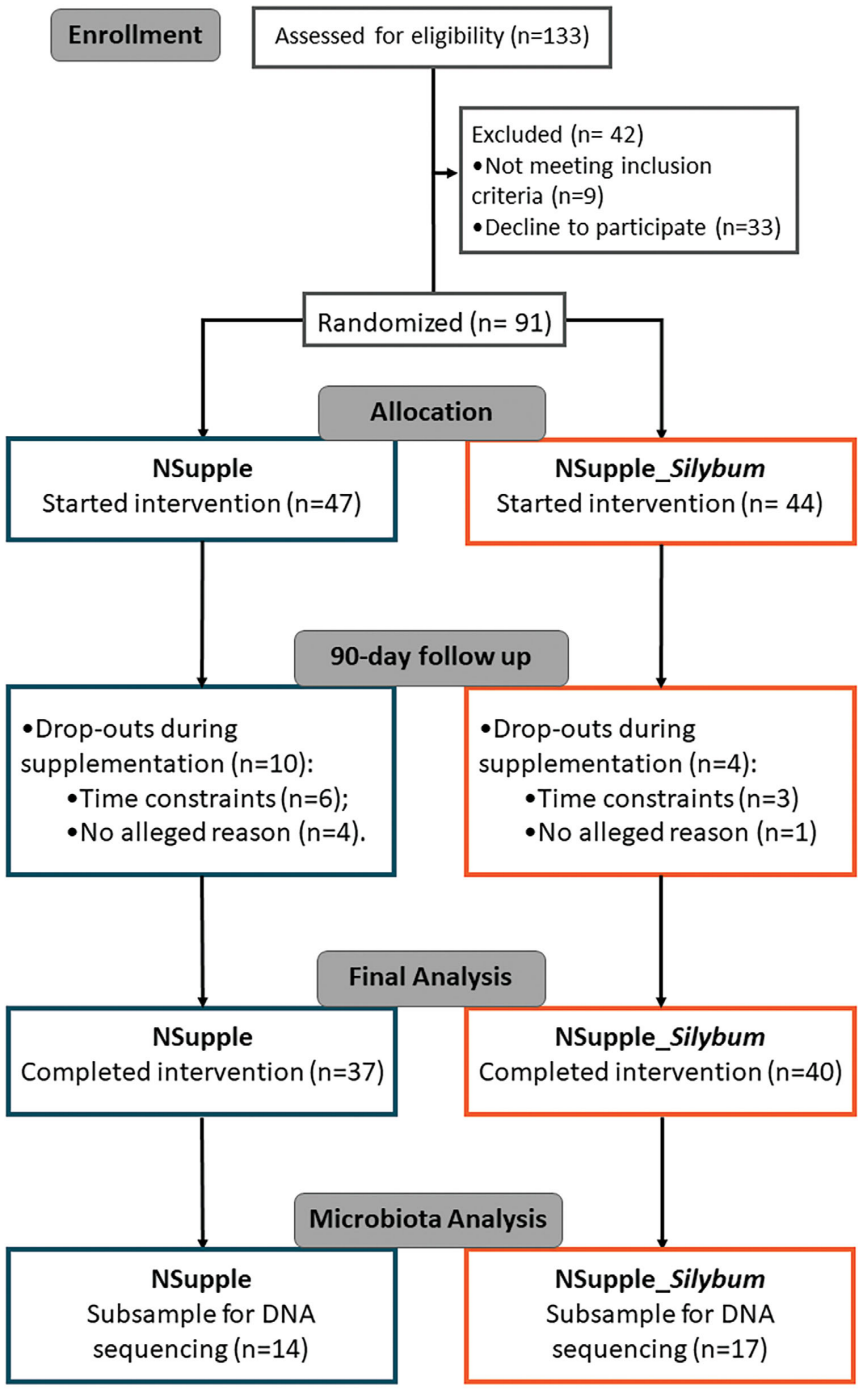
Consolidated Standards of Reporting Trials (CONSORT) flowchart describing the experimental design carried out.

Volunteers who met the inclusion criteria underwent stratified randomization based on age, sex, and body mass index (BMI). The trial's progression is detailed in the CONSORT (Consolidated Standards of Reporting Trials) flow chart (Figure 1). Initially, 133 individuals responded to the recruitment call. Of these, 42 withdrew before randomization due to not meeting the inclusion criteria (*n* = 9) or declining further participation (*n* = 33). Thus, 91 participants were randomized into two groups: NSupple (*n* = 47) and NSupple_*Silybum* (*n* = 44) at T0. During the 90‐day supplementation period, 10 participants from the NSupple group were excluded due to time constraints (*n* = 6) or unspecified reasons (*n* = 4). Similarly, in the NSupple_*Silybum* group, 14 participants withdrew due to time constraints (*n* = 4) or unspecified reasons (*n* = 10). By the end of the trial, 37 participants from the NSupple group and 40 participants from the NSupple_*Silybum* group completed the 90 days of supplementation. Blood and fecal samples were collected from all participants at both T0 and T90. For gut microbiota analysis, a subsample calculation was conducted as described in the Statistical Analysis section. Volunteers were randomly selected for DNA sequencing, resulting in 14 individuals from the NSupple group and 17 from the NSupple*_Silybum* group. All participants provided informed consent before participating in the study.

### 2.5 Volunteers’ Dietary Intake, Stool Consistency, and Anthropometrics

Dietary intake data were collected at baseline (T0) and after 90 days of supplementation (T90) using a 3‐day food record, which was analyzed with DietPro software (version 6.1). To account for seasonal and weekly variations, participants were instructed to complete non‐consecutive 3‐day records, ensuring that at least 1 day included a weekend or holiday. To improve portion size accuracy, participants recorded food quantities using multiples of common household tableware. A standardized protocol included a detailed manual outlining the food recording procedure. Participants received training based on this manual, and dietitians reviewed the records for unclear descriptions, errors, omissions, or questionable entries. Any discrepancies were clarified with the participants to ensure data accuracy [36].

Stool consistency was assessed using the “Bristol Stool Form Scale” (BSFS) [37], a visual chart with accompanying text that classifies stools into seven types: (1) separate hard lumps, like nuts; (2) sausage‐shaped but lumpy; (3) like a sausage or snake with cracks on its surface; (4) like a sausage or snake, smooth and soft; (5) soft blobs with clear‐cut edges; (6) fluffy pieces with ragged edges, mushy; (7) watery, no solid pieces. Types 1 and 2 indicate constipation, Types 3, 4, and 5 indicate normal consistency, and Types 6 and 7 indicate diarrhea. For this study, the Portuguese‐validated version of the BSFS was used [38].

Anthropometric measurements were taken at T0 and T90. Body mass and height were measured using the Body Composition Scale 2 (Xiaomi Mi, Beijing, China). Neck, waist, hip, and iliac crest circumferences were measured with a plastic tape measure, and the waist‐to‐height ratio (WHtR) was calculated. The BMI was calculated as body mass (kg)/height (m) [2], and the waist‐to‐hip ratio (WHR) was provided in the supplementary data (Table S1).

### 2.6 Plasma Analysis

Blood samples were collected at baseline (T0) and after 90 days (T90) between 7:00 a.m. and 9:00 a.m. for analysis of albumin, total cholesterol, cortisol, creatinine, alkaline phosphatase, glycemia, IgM, LDL‐c, total protein, ALT (alanine transaminase), and TSH (thyroid‐stimulating hormone). The AST/ALT ratio (aspartate transaminase/alanine transaminase ratio), cortisol/C‐RP ratio (C‐reactive protein), and atherogenic index were calculated. Additionally, insulin, HOMA‐IR (Homeostatic Model Assessment for Insulin Resistance), C‐reactive protein (C‐RP), cholesterol fractions (HDL‐c: high‐density lipoprotein‐cholesterol, VLDL‐c: very low‐density lipoprotein‐cholesterol, non‐HDL‐c: nonhigh‐density lipoprotein‐cholesterol), triglycerides, IgG (Immunoglobulin G), IgA (Immunoglobulin A), AST (aspartate transaminase)s, gamma‐GT (gamma‐glutamyl transferase), and thyroxine were analyzed and are presented in Supporting Information (Table S1). These analyses were conducted by the *“Fleury Medicina e Saúde”* Laboratory.

Plasma samples were evaluated using a “cytometric bead array” (CBA) test to quantify cytokines and chemokines. Beads, standards, reagents, and plasma samples were prepared according to the manufacturer's guidelines. Results were obtained using the FACS Canto II flow cytometer (BD Biosciences, USA) and commercially available kits (BD Cytometric Bead Array) for “Human Chemokine” and “Human Inflammatory Cytokines” (BD Biosciences, USA). Standard dilution methods were applied for cytokine standards. Calibration of the flow cytometer with cytometer setup beads was performed before the assay. Data analysis was conducted using CBA analysis software (SoftFlow, Pecs, Hungary), with results presented in pg/mL.

### 2.7 Silymarin Plasma Concentration

**FIGURE 2:**
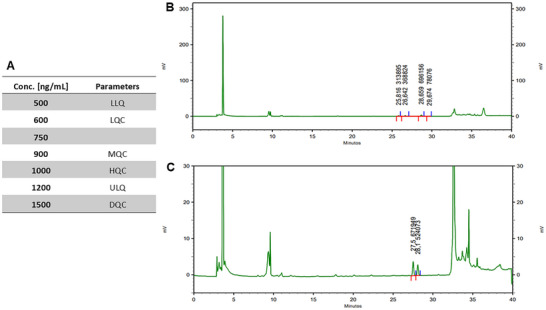
Plasma silymarin detection analysis: (A) Chromatogram of the lower limit of quantification (LLQ) sample, (B) chromatogram of the plasma sample from NSupple_*Silybum* group, where only the internal standard was added for analysis, and (C) concentrations used for the construction of the analytical curve and the parameters evaluated with these concentrations.

To evaluate the absorption rate of silymarin, its quantification in plasma was conducted, as silymarin extract was the primary distinguishing factor between the experimental groups. The method validation, essential for adhering to bioanalytical standards [39], was completed using flubendazole as an internal standard (IS) and silymarin as an analytical standard. Flubendazole was chosen due to its appropriate retention time and lack of peak overlap with silymarin. Both standards were prepared by dissolving 3 mg of flubendazole and 10 mg of silymarin in HPLC‐grade acetonitrile, achieving a final concentration of 4.5 µg/mL. The calibration curve was established using seven concentration points, ranging from 500 to 1500 ng/mL, including high, medium, and low‐quality control (HQC, MQC, and LQC, respectively), as well as dilution quality control (DQC) and the upper and lower limits of quantification (ULQ and LLQ) as shown in Figure 2A. Key parameters such as linearity, selectivity, matrix effect, precision, accuracy, and sample stability were assessed.

The analytical method involved plasma stored at −80°C, with protein precipitation and analyte extraction performed using cold HPLC‐grade acetonitrile in a 1:1 plasma ratio. After centrifugation, the supernatant was filtered. The calibration curve was prepared by adding 50 µL of the internal standard and 100 µL of silymarin in acetonitrile to 150 µL of plasma. Patient plasma samples were similarly prepared and analyzed. The analyses employed high‐performance liquid chromatography (HPLC) with DAD detection, using a mobile phase of water with 0.1% formic acid and methanol with 0.1% formic acid. The gradient started with 10% organic solvent and increased systematically until reaching 100% at minute 31, followed by column washing and stabilization. Chromatograms were processed with EZChrom Elite software and analyzed at 289 nm. The method proved reliable for quantifying silymarin in human plasma above 500 ng/mL. However, silymarin was not detected in the patient samples, despite improvements in other plasma parameters (Figure 2B,C).

### 2.8 Questionnaires for Sleep, Mood, and Quality of Life Characterization

The subjective sleep quality was assessed using the “Brazilian Portuguese Version of the Mini‐Sleep Questionnaire” (MSQ‐BR) [40]. The “Epworth Sleepiness Scale” (ESS) [41] was applied to measure general daytime sleepiness levels. To determine the chronotype of the volunteers, the Portuguese version of the Horne and Ostberg Morningness‐Eveningness Questionnaire was used [42]. The “Pittsburgh Sleep Quality Index” (PSQI) [43] was applied to evaluate sleep quality and disturbances over 1 month. To assess the participants’ perception of quality of life, the “World Health Organization Quality of Life Instrument‐Short Form” (WHOQoL‐BREF) [44] was administered. “The Brunel Mood Scale” (BRUMS) [45] was used as a validated instrument to screen the volunteers' mood perception. The Brazilian Economic Classification Criterion (ABEP) was applied to assess the socioeconomic status by evaluating household characteristics and the education level of the household head. Scores categorize households into economic classes (A to E), estimating purchasing power and supporting social and market research [46].

### 2.9 Microbiome Analysis

#### 2.9.1 Sample Collection and Genomic Extraction

Participants collected approximately 1 g of feces and placed it in a guanidine stool preservation medium to promptly preserve the microbiome. Samples were transported under controlled temperatures (2°C–8°C) and stored at −80°C [47]. Genomic material was extracted from about 0.25 g of feces using the DNeasy PowerSoil Kit (Qiagen, Germantown, MD, USA) and stored at −20°C until library preparation.

#### 2.9.2 Library Preparation and Sequencing and Bioinformatic Analysis

Library preparation and sequencing procedures followed the methods detailed by Nehmi‐Filho et al. [12]. Briefly, 16S rRNA (V4 region) sequences for prokaryotic community analysis were amplified and sequenced using the 515F/806R [48] primer set. Sequencing was performed on the Ion Chef System and Ion S5 platform according to the manufacturer's instructions (Thermo Fisher Scientific, Waltham, Massachusetts, USA).

The bioinformatic analysis, as described by Nehmi‐Filho et al. [12], involved preprocessing and diversity estimation using Quantitative Insights Into Microbial Ecology (QIIME 2) version 2020.11 [49]. An average of 42,577 sequences per sample were denoised with DADA2, generating 2561 amplicon sequence variants (ASVs) [50]. Alpha and beta diversity metrics were calculated after rarefying samples to 14,729 sequences each [51]. Taxonomic classification of ASVs used the Q2‐feature‐classifier [52] with a naive Bayes classifier against the Greengenes 13_8 99% OTUs (operational taxonomic units) reference sequences [53]. Microbiota community composition was summarized at species, genera, families, orders, classes, and phyla levels.

Alpha diversity metrics included Chao1, Simpson, OTUs, Pielou's evenness, Shannon diversity, and Faith's phylogenetic diversity. Beta diversity metrics included Jaccard distance, Bray–Curtis distance, and unweighted and weighted UniFrac distances. Heatmap visualization was used for microbiome data interpretation at the phyla, genera, and species levels, and differential abundance analysis was performed using the DESeq2 package in R (version 4.3.1).

### 2.10 Statistical Analysis

The sample size was determined using G*Power 3.1 software (Faul et al. 54), assuming a *t*‐test (Means: Difference between two dependent means‐matched pairs) with (*α*) level of 0.05, a power (*β*) of 0.95, and an effect size (success rate) of 0.5. The calculated total sample size required was 54 volunteers. Considering potential dropouts, we increased the sample size by 20%, resulting in an ideal sample size of at least 65 volunteers to ensure data reliability. Continuous data were classified as parametric or nonparametric based on the Shapiro–Wilks and Smirnov–Kolmogorov tests; outliers were excluded by the Grubbs’ test. Continuous parametric data were shown as mean ± standard deviation, and nonparametric as median and interquartile range. Continuous parametric variables were performed using Student *t*‐test, and for nonparametric variables, the Wilcoxon test was applied. Categorical variables’ results were expressed in absolute and relative frequencies and were analyzed using the Chi‐Square test or Fisher's Exact test. Linear and logistic regression tests were performed for continuous and categorical variables, respectively, to verify all variables associated with the outcomes of interest investigated in this study. For the linear regressions, the magnitudes of the differences between the baseline and final results were stipulated for each variable of interest in comparison with the dependent variable. For the logistic regressions, the normality thresholds were verified for each variable of interest when present and researched in the scientific literature, or when absent, the lower and upper thresholds were defined to the 50% quartile for all variables to be investigated. Analyses were performed using STATA 16‐SE (Stata Corp. LCC, College Station, TX, USA) and GraphPad Prism 9.0 (GraphPad Software, La Jolla, CA, USA) software.

A subsample for microbiota 16S rRNA (V4 region) sequences was determined as the representative portion of the larger sample used for analysis [55]. The subsample was randomly selected to ensure it accurately reflects the characteristics of the whole population while still obtaining accurate and reliable results. The genera that were differentially represented between supplement groups were determined using the R (4.3.1) package DESeq2 (1.42.0). Differential gene expression analysis based on the negative binomial distribution [56]. To determine the taxonomic characteristics most likely to explain differences between periods and supplement groups, we employed the algorithm linear discriminant analysis effect size (LEfSe 1.1.2) [57]. For all analyses, significance was determined as *p* ≤ 0.05.

## 3 Results

### 3.1 Anthropometric Data, Diet Intake, and Stool Consistency

The sample homogeneity was confirmed by the similarity in demographic characteristics such as age, height, and male/female ratio between groups. Regarding anthropometric data, the NSupple_*Silybum* group showed significant body weight reduction after 90 days of supplementation. This group also exhibited neck and waist circumference reductions at the waist circumference at the iliac crest (WC‐IC). Conversely, the NSupple group experienced reduced waist circumference at the waist circumference at the mid‐abdomen (WC‐mid), hip circumference, and waist‐to‐height ratio (WHtR) post‐supplementation. Interestingly, despite the reduction in anthropometric measures, the NSupple groups showed higher caloric intake post‐supplementation, with the NSupple_*Silybum* group also displaying increased carbohydrate intake. Other dietary data evaluated did not change after the 90 days of supplementation. According to the BSFS, the NSupple*_Silybum* group reported improved stool consistency and a reduction in the prevalence of constipation (Types 1 and 2) after supplementation, as shown in Table 2. The BMI and waist‐to‐hip ratio (WHR) were evaluated and showed no significant changes, as presented in Supporting Information (Table S1).

**TABLE 2:** Demographic and anthropometric characterization, dietary intake, and Bristol Stool Form Scale classification.

Demographics		
	NSupple	NSupple_*Silybum*
Sample size	37	40
Sex (M/F)	11/26	10/30
Age (years)	55 ± 5	54 ± 6
Brazilian Economic Classification Criterion % (*n*)		
A	2.70 (1)	2.50 (1)
B	59.46 (22)	60 (24)
C	35.14 (13)	35 (14)
D	2.70 (1)	2.50 (1)
E	0 (0)	0 (0)
Anthropometrics		
Height (m)	1.63 ± 0.017	1.64 ± 0.013

	NSupple			NSupple_*Silybum*		
	T0	T90		T0	T90	
	Mean ± SD	Mean ± SD	*p*	Mean ± SD	Mean ± SD	*p*
Body weight (kg)	74.42 ± 2.14	74.19 ± 1.97	*∔*	74.51 ± .2.31	74.19 ± 2.29	0.042
BMI (kg/m^2^)	27.97 ± 0.66	27.92 ± 0.63	*∔*	27.35 ± 0.63	27.48 ± 0.65	*∔*
Neck (cm)	36.09 ± 0.58	35.95 ± 0.63	*∔*	36.23 ± 0.54	36.09 ± 0.58	0.0285
WC‐mid (cm)	91.91 ± 1.64	90.73 ± 1.58	0.028	91.48 ± 1.82	90.37 ± 1.84	*∔*
Hip (cm)	106.60 ± 1.39	105 ± 1.67	0.050	104.6 ± 1.25	104.2 ± 1.38	*∔*
WC‐IC (cm)	100.60 ± 1.44	100.8 ± 1.40	*∔*	101.7 ± 1.47	99.56 ± 1.40	0.0121
WHtR	0.56 ± 0.01	0.56 ± 0.01	*∔*	0.56 ± 0.01	0.55 ± 0.01	0.0375

Dietary intake						
	NSupple			NSupple_*Silybum*		
	T0	T90		T0	T90	
	Mean ± SD	Mean ± SD	*p*	Mean ± SD	Mean ± SD	*p*
Energy (Kcal)	1724 ± 108.9	1747 ± 110.3	0.0001	1782 ± 75.06	1887 ± 127.7	—
Carbohydrates (g)	235.3 ± 13.12	228.4 ± 17.76	—	226.3 ± 12.10	270.7 ± 17.57	0.0059
Fiber (g)	12.91 ± 1.661	12.04 ± 1.33	—	12.18 ± 1.67	11.40 ± 1.29	—
Lipids (g)	55.83 ± 5.62	58.64 ± 5.11	—	61.40 ± 5.88	65.60 ± 7.98	—
Proteins (g)	75.570 ± 7.31	76.99 ± 7.59	—	68.33 ± 5.01	73.99 ± 6.77	—

Bristol Stool Form Scale (BSFS)						
	NSupple			NSupple_*Silybum*		
	T0	T90	*p*	T0	T90	*p*
	% (*n*)	% (*n*)		% (*n*)	% (*n*)	
Types 1 and 2	19 (7)	19 (7)		27.5 (11)	22.5 (9)	
Types 3, 4, and 5	68 (25)	65 (24)		62.5 (25)	65 (26)	
Types 6 and 7	14 (5)	16 (6)		10 (4)	12.5 (5)	
						0.0093

Abbreviations: WC‐IC: waist circumference in iliac crest; WC‐mid: waist circumference in middle abdomen; WHtR: waist‐to‐height ratio.

### 3.2 Biochemistry and Cytokines in Plasma

Biochemical analysis of the volunteers' plasma before and after 90 days of supplementation is presented in Table 3. The NSupple group showed increased albumin and total cholesterol levels, while glycemia, total proteins, TSH, the cortisol/C‐RP ratio, and the atherogenic index decreased post‐supplementation. In the NSupple*_Silybum* group, there was an increase in alkaline phosphatase and glycemia, along with decreases in IgM, LDL‐cholesterol (low‐density lipoprotein‐cholesterol), total protein, and alanine aminotransferase (ALT) levels. Additionally, both groups exhibited reduced cortisol levels and the AST/ALT ratio post‐supplementation (Table 3). Other biochemical parameters did not show significant changes between groups and time points, as displayed in Table S1 in the Supporting Information.

**TABLE 3:** Serum parameters in the study population before and after the supplementation.

	NSupple			NSupple_*Silybum*		
	T0	T90		T0	T90	
	Mean ± SD	Mean ± SD	*p*	Mean ± SD	Mean ± SD	*p*
Albumin (g/dL)	4.44 ± 0.05	4.50 ± 0.05	0.0257	4.55 ± 0.03	4.55 ± 0.04	—
Total Cholesterol (mg/dL)	218.80 ± 5.81	228.8 ± 7.58	0.0463	205.9 ± 7.13	199.8 ± 6.35	—
Cortisol (ug/dL)	14.81 ± 0.78	12.48 ± 0.79	< 0.0001	13.32 ± 0.69	11.49 ± 0.78	0.0255
Creatinine (mg/dL)	0.84 ± 0.03	0.81 ± 0.03	—	0.81 ± 0.03	0.77 ± 0.03	0.0232
Alkaline phosphatase (U/L)	76.28 ± 3.42	76.5 ± 3.51	—	73.77 ± 3.56	76.94 ± 3.26	0.0235
Glycemia (mg/dL)	89.29 ± 1.70	86.76 ± 1.85	0.0262	86.93 ± 1.38	90.87 ± 1.36	0.002
IgM (mg/dL)	112.9 ± 7.79	105.8 ± 7.45	—	111.8 ± 8.13	101.4 ± 6.75	0.0001
LDL‐C (mg/dL)	133.9 ± 4.81	139.5 ± 5.17	—	128.8 ± 6.21	121.6 ± 4.96	0.0241
Total protein (g/dL)	7.156 ± 0.08	6.97 ± 0.08	0.0018	7.146 ± 0.05	6.96 ± 0.06	0.0013
ALT (U/L)	10.03 ± 0.82	9.55 ± 0.65	—	9.84 ± 0.68	9.01 ± 0.86	0.0023
TSH (mUI/L)	3.18 ± 031	2.61 ± 0.23	0.0014	2.809 ± 0.23	2.724 ± 0.25	—
AST/ALT ratio	2.33 ± 0.16	1.98 ± 0.15	0.0331	2.296 ± 0.15	1.91 ± 0.13	< 0.0001
Cortisol/C‐RP ratio	87.8 ± 8.40	74.71 ± 9.13	0.0354	92.83 ± 11.56	87.89 ± 12.86	—
Atherogenic index	0.44 ± 0.06	0.36 ± 0.05	0.0406	0.42 ± 0.04	0.44 ± 0.05	—

Magnitude of difference					
	NSupple		NSupple_*Silybum*		
	Mean ± SD	Median (Q1; Q3)	Mean ± SD	Median (Q1; Q3)	*p*
Δ non‐HDL‐c (mg/dL)	10.57 ± 35.76	6.00 (−3.00; 19.00)	−17.32 ± 67.91	3.00 (−27.00; 9.00)	0.030
Δ total Cholesterol (mg/dL)	11.97 ± 33.92	7.00 (−2.00; 23.00)	−17.88 ± 71.55	−4.50 (−29.00; 8.00)	0.010
Δ LDL‐c (mg/dL)	−1.11 ± 40.63	6.00 (−9.00; 16.00)	−7.39 ± 20.67	−5.00 (−19.00; 9.00)	0.041

Abbreviations: ALT: alanine aminotransferase; AST: aspartate aminotransferase; C‐RP: C‐reactive protein; IgM: Immunoglobulin M; TSH: thyroid‐stimulating hormone.

To assess the impact of each supplement on biochemical parameters, the magnitude of difference was analyzed to compare the effects between the two groups. The NSupple_*Silybum* group exhibited a significant reduction in Δ total cholesterol, Δ non‐HDL cholesterol, and Δ LDL cholesterol, attributable to the supplement compared to the NSupple group (Table 3).

**FIGURE 3:**
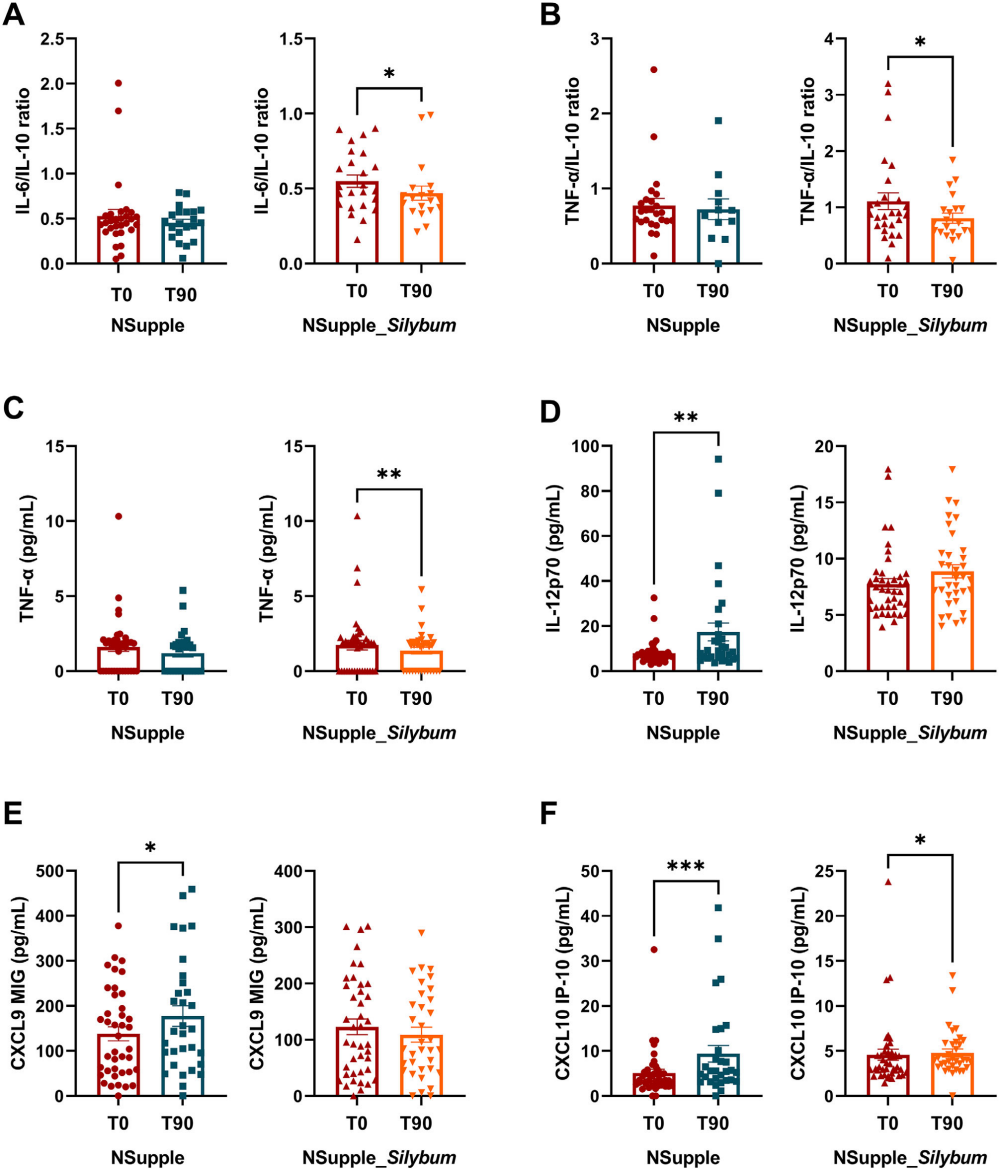
Serum inflammatory parameters evaluated before and after supplementation (A) IL‐6/IL‐10 ratio, (B) TNF‐α/IL‐10 ratio, (C) TNF‐α, (D) IL‐12p70, (E) CXCL9 MIG, (F) CXCL10 IP‐10. Values are expressed as pg/mL (mean ± standard deviation). *p < 0.05, **p < 0.01, ***p < 0.001, ***p < 0.001.

Post‐supplementation cytokine evaluation revealed a reduction in the IL‐6/IL‐10 ratio (interleukin‐6 / interleukin‐10 ratio) (Figure 3A), TNF‐α/IL‐10 ratio (tumor necrosis factor‐alpha / interleukin‐10 ratio) (Figure 3B), TNF‐α (tumor necrosis factor‐alpha) (Figure 3C), and CXCL10 IP10 (chemokine interferon‐gamma‐inducible protein 10) (Figure 3F) levels in the NSupple_*Silybum* group. Conversely, the NSupple group showed increased levels of IL‐12p70 (Interleukin‐12p70) (Figure 3D), CXCL9 MIG (monokine induced by interferon‐gamma) (Figure 3E), and CXCL10 I10 (Figure 3F) after the 90‐day supplementation. Levels of IL‐1β (Interleukin‐1 beta), IL‐6 (Interleukin‐6), IL‐8 (interleukin‐8), IL‐10 (Interleukin‐10), and CCL5/RANTES (C‐C motif ligand 5 5 Regulated‐on‐Activation‐Normal‐T‐cell_Expressed‐and‐Secreted) did not show significant changes, as shown in Supporting Information (Table S2).

### 3.3 Sleep, Mood, and Quality of Life

The Horne and Ostberg Morningness‐Eveningness Questionnaire indicated that the majority of volunteers were classified as either intermediate or moderately evening chronotypes, with a balanced distribution between groups, as shown in Table 4. The ESS scores improved in the NSupple group, indicating reduced sleepiness. The Pittsburgh Sleep Quality Index (PSQI) (C1) Sleep Quality component also improved post‐supplementation exclusively in the NSupple group (Table 4). The Mini‐Sleep Questionnaire (MSQ‐BR) and BRUMS did not show significant differences between groups or evaluation times (Table S3). The WHO Quality of Life questionnaire (WHOQoL‐BREF) revealed an increase in overall quality of life and general health in the NSupple_*Silybum* group, while the psychological domain in WHOQoL‐BREF improved in the NSupple group post‐supplementation (Table 4).

**TABLE 4:** Chronotype, sleep quality, daytime sleepiness, and quality of life in the study population before and after the supplementation.

Horne and Ostberg Morningness‐Eveningness Questionnaire—Chronotype		
Group	NSupple	NSupple_*Silybum*
	% (*n*)	% (*n*)
Definitely morning	3% (*n* = 1)	—
Moderately morning	—	7.5% (*n* = 3)
Intermediate	46% (*n* = 17)	45% (*n* = 18)
Moderately evening	30% (*n* = 11)	37.5% (*n* = 15)
Definitely evening	21% (*n* = 8)	10% (*n* = 4)

	T0		T90			T0		T90		
	Mean ± SD	CI 95%	Mean ± SD	CI 95%	*p*	Mean ± SD	CI 95%	Mean ± SD	CI 95%	*P*
Epworth Sleepiness Scale (ESS)										
ESS Total score	10.37 ± 0.81	8.73–12.01	9.28 ± 0.89	7.47–11.08	0.0143	10.00 ± 0.77	8.45–11.55	9.56 ± 0.95	7.64–11.49	—
Pittsburgh Sleep Quality Index (PSQI)										
PSQI Global score	6.14 ± 0.41	5.32–6.96	5.24 ± 0.55	4.14–6.35	—	7.06 ± 0.53	5.99–8.13	6.61 ± 0.63	5.33–7.88	—
(C1) Sleep quality	1.19 ± 0.12	0.94–1.43	0.92 ± 0.13	0.66–1.18	0.0325	1.24 ± 0.14	0.95–1.53	1.16 ± 0.13	0.89–1.43	—
(C2) Sleep latency	1.14 ± 0.14	0.86–1.42	0.76 ± 0.14	0.47–1.04	—	1.33 ± 0.17	0.98–1.67	1.08 ± 0.16	0.76–1.40	—
(C3) Sleep duration	1.16 ± 0.15	0.86–1.47	1.14 ± 0.16	0.81–1.46	—	1.28 ± 0.14	1.01–1.56	1.32 ± 0.20	0.90–1.73	—
(C4) Sleep efficiency	0.41 ± 0.12	0.17–0.64	0.52 ± 0.15	0.21–0.82	—	0.49 ± 0.14	0.21–0.77	0.58 ± 0.15	0.27–0.89	—
(C5) Sleep disturbance	1.19 ± 0.09	1.01–1.37	0.97 ± 0.07	0.83–1.12	—	1.15 ± 0.09	0.96–1.34	1.10 ± 0.11	0.89–1.32	—
(C6) Sleep medication	0.33 ± 0.14	0.05–0.61	0.24 ± 0.14	0.034–0.52	—	0.61 ± 0.16	0.29–0.92	0.53 ± 0.18	0.17–0.88	—
(C7) Daytime dysfunction	0.74 ± 0.10	0.54–0.95	0.71 ± 0.13	0.44–0.96	—	0.98 ± 0.13	0.71–1.25	0.84 ± 0.10	0.63–1.05	—
WHO Quality of Life‐BREF Overall QoL and general health	3.57 ± 0.11	3.35–3.79	3.67 ± 0.12	3.43–3.91	—	3.38 ± 0.11	3.16–3.60	3.65 ± 0.11	3.40–3.85	0.006
Physical domain	3.78 ± 0.09	3.6–3.96	3.77 ± 0.08	3.62–3.93	—	3.61 ± 0.09	3.42–3.80	3.73 ± 0.10	3.53–3.93	*∔*
Social relationships domain	3.71 ± 0.08	3.54–3.88	3.67 ± 0.09	3.49–3.84	—	3.58 ± 0.11	3.35–3.81	3.50 ± 0.11	3.27–3.73	—
Environment domain	3.49 ± 0.07	3.35–3.64	3.43 ± 0.08	3.27–3.59	—	3.51 ± 0.08	3.35–3.68	3.57 ± 0.08	3.42–3.72	—
Psychological domain	3.68 ± 0.07	3.53–3.83	3.69 ± 0.07	3.54–3.84	0.0283	3.61 ± 0.08	3.43–3.78	3.65 ± 0.08	3.49–3.82	—

### 3.4 Gut Microbiota Reshaping

**FIGURE 4:**
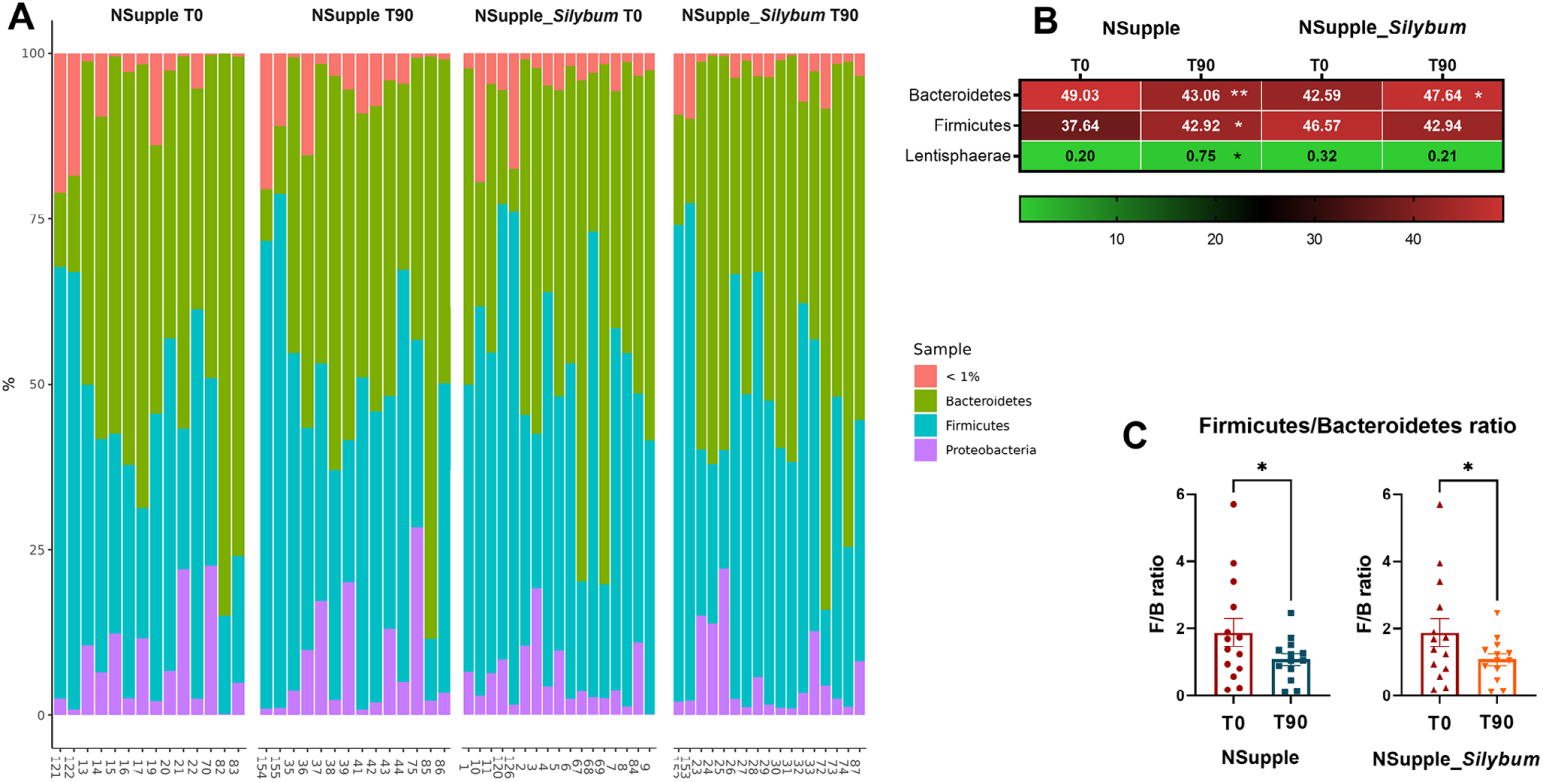
Phyla composition of gut microbiota after 90 days of supplementation. (A) Abundance profile of the phyla. (B) Heatmap of microbial phyla abundance with statistical significance. (C) Firmicutes/Bacteroidetes ratio (F/B ratio) before [T0] and after [T90] supplementation. Values are expressed as the percent of relative abundance (mean ± standard deviation). *p < 0.05, **p < 0.01, ***p < 0.001.

The volunteers’ gut microbiota primarily comprised the phyla Firmicutes, Bacteroidetes, and Proteobacteria, as depicted in Figure 4A. After 90 days of supplementation, the NSupple group exhibited a reduction in Bacteroidetes and an increase in Firmicutes and Lentisphaerae phylum abundance, while the NSupple_*Silybum* group showed an increase in Bacteroidetes with no significant changes in other phyla (Figure 4B). Both groups demonstrated reduced Firmicutes/Bacteroidetes (F/B) ratio (Figure 4C).

**FIGURE 5:**
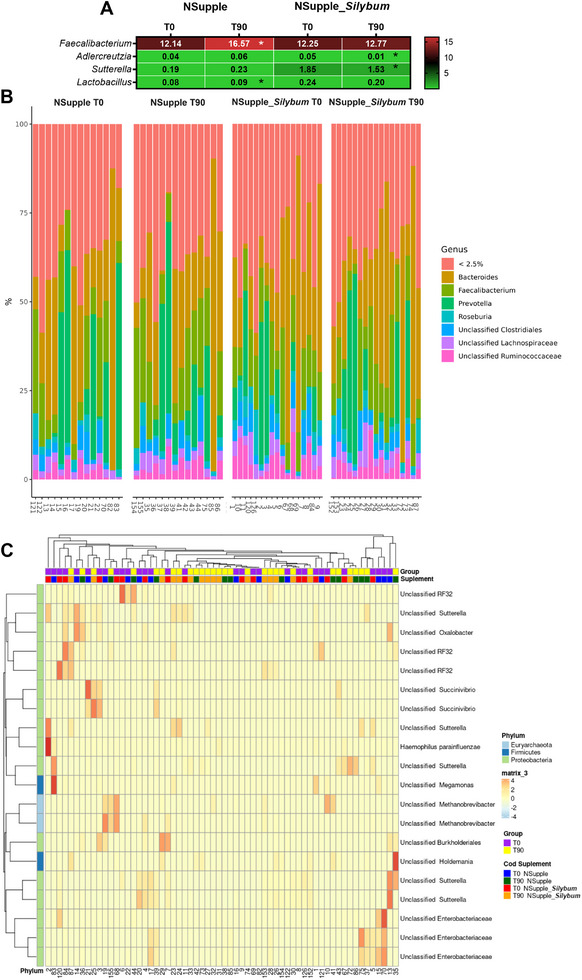
Genera composition of the gut microbiota in volunteers after 90 days of supplementation. (A) Heatmap of microbial genera abundance with statistical significance. (B) Abundance profile of the genera. (C) Heatmap depicting the taxonomic distribution of volunteers’ microbiome by LEfSe (linear discriminant analysis effect size). Values are expressed as the percent of relative abundance (mean ± standard deviation). *p < 0.05, **p < 0.01, ***p < 0.001.

At the genus level, the most abundant genera identified were *Bacteroides, Faecalibacterium, Prevotella, Roseburia*, and unclassified *Clostridiales, Lachnospiraceae*, and *Ruminococcaceae* (Figure 5B). Supplementation effects revealed that the NSupple group had an increase in the genera *Faecalibacterium* and *Lactobacillus*, whereas the NSupple_*Silybum* group showed a reduction in *Adlercreutzia* and *Sutterella* (Figure 5A). The heatmap in Figure 5C illustrates the main phyla and genera with differential representation between the supplemented groups and at different time points during supplementation.

**FIGURE 6:**
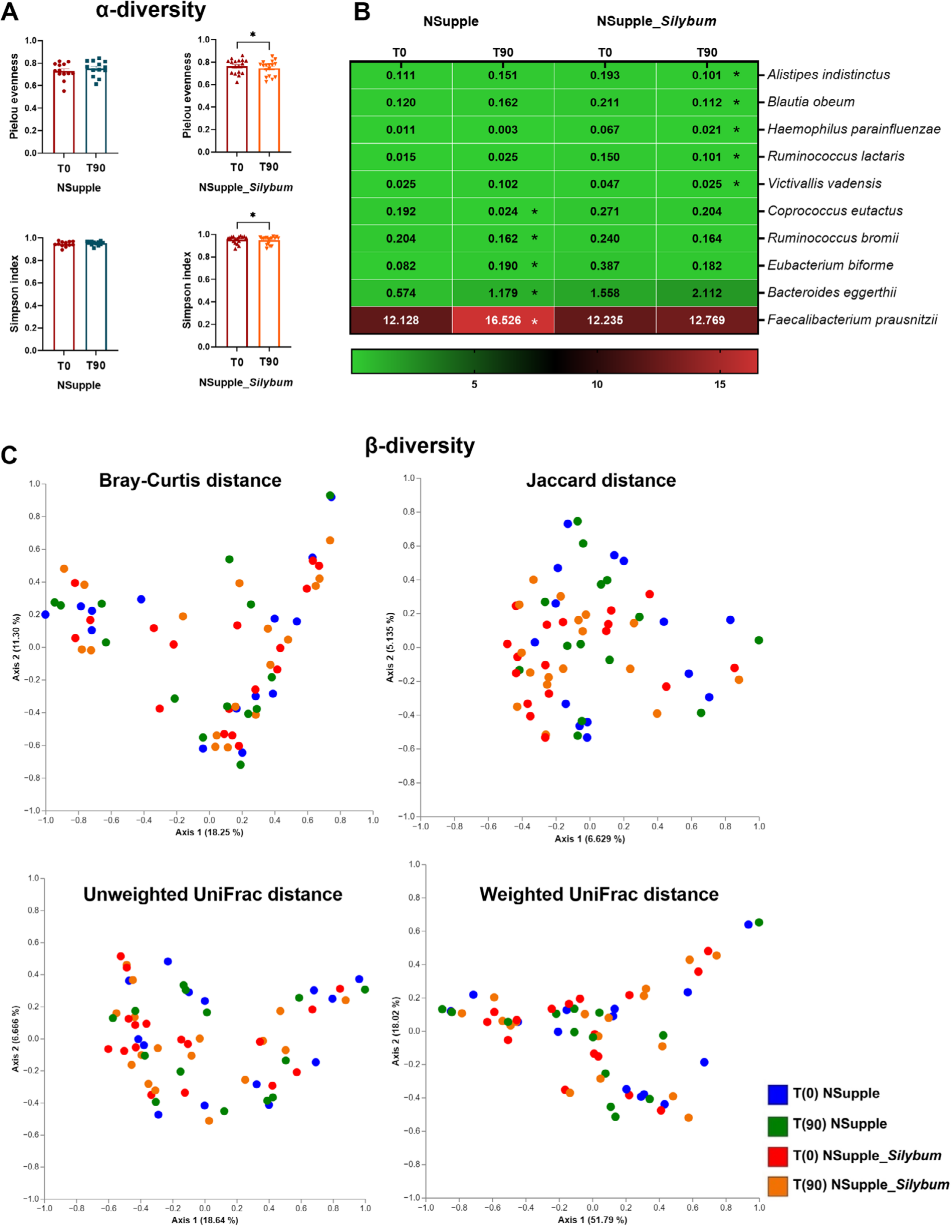
Diversity indices and species composition of the gut microbiota in volunteers after 90 days of supplementation. (A) Alpha (*α*) diversity indices for species richness (Shannon) and phylogenetic diversity (Faith's PD). (B) Heatmap of microbial species abundance with statistical significance. (C) Beta (β)‐diversity indices including Jaccard distance, Bray–Curtis distance, unweighted UniFrac, and weighted UniFrac distances before [T0] and after 90 days [T90] of supplementation. Values are expressed as the percent of relative abundance (mean ± standard deviation). *p < 0.05, **p < 0.01, ***p < 0.001.

Microbiota indices of alpha (*α*) and beta (*β*)‐diversity were also assessed. The alpha diversity indices Pielou's evenness, and Simpson index (Figure 6A) were reduced in the NSupple_*Silybum* group post‐supplementation. Other alpha diversity indices, including Chao1, Faith's Phylogenetic Diversity, Shannon index, and Observed features, did not differ significantly and are presented in Figure S2. Beta diversity indices—Bray–Curtis distance, Jaccard distance, unweighted UniFrac distance, and weighted UniFrac distance—showed no significant differences between groups and time points visually represented by the Principal Coordinate Analysis Plot (PCoA) (Figure 6C).

The heatmap in Figure 6B highlights the species modulated by supplementation. Specifically, the NSupple_*Silybum* group exhibited reductions in *Alistipes indistinctus, Blautia obeum, Haemophilus parainfluenzae, Ruminococcus lactaris*, and *Victivallis vadensis* post‐supplementation. Additionally, the NSupple group showed a decrease in *Coprococcus eutactus* and *Ruminococcus bromii* but an increase in *Bacteroides eggerthii* and *F. prausnitzii* post‐supplementation.

### 3.5 Variables Association Post‐Supplementation

Please improve the text for clarity, keeping the scientific tone and conciseness: The variable associations shown in Table 5 highlight the association between improvement of sleep patterns and inflammatory markers in the NSupple_*Silybum* group. Linear regression analysis reveals a correlation between the WHtR and improvement in ESS scores within this group. Similarly, the sleep quality measured by the MSQ score was associated with WHtR in multiple linear regression analysis. Also, the IL‐10 plasma level was associated with the improvement of (C4) sleep efficiency in the PSQI score shown in Table 5.

**TABLE 5:** Regression analysis of data association after supplementation.

Multiple linear regression						
Group			Coef.	IC95% min	IC95% max	*p*
NSupple_*Silybum*	WHtR	ESS	0.003	0.000	0.006	0.023
		MSQ	0.0036	0.0009	0.0063	0.012
	IL‐10	(C4) Sleep efficiency	−0.4807	−0.8784	−0.0830	0.024

Multiple logistic regression						
Group			Coef.	IC95% min	IC95% max	*p*
NSupple_*Silybum*	Cortisol	↓(C3) Sleep duration	0.154	0.025	0.950	0.044
	IL‐10	↓ MSQ	0.154	0.036	0.651	0.011
	L‐12p70	↓ (C1) Sleep quality	7.000	1.098	44.608	0.039
		↓ PSQI total	5.333	1.175	24.213	0.030

Multiple logistic regression microbiota subsample						
Group			Coef.	IC95% min	IC95% max	*p*
NSupple_*Silybum*	Body weight	Lentisphaerae	0.0431	0.0019	0.9584	0.047
		*R. ruminococcus*	0.0728	0.0056	0.9477	0.045
	L‐12p70	*Phascolarctobacterium*	15.00	1.0307	218.3001	0.047
	CXCL10 IP10	*R. ruminococcus*	24.00	1.7412	330.8045	0.018

Additionally, logistic regression identifies a significant association between cortisol levels and improvements in sleep duration (C3), as well as between IL‐12p70 levels and enhancements in both sleep quality (C1) and overall sleep domain patterns, as measured by PSQI scores. Furthermore, the anti‐inflammatory cytokine IL‐10 was significantly correlated with improved sleep quality as reflected in the MSQ scores (Table 5). Evaluating the microbiota subsample associations, the body weight was associated with the Lentisphaerae phylum and *R. ruminococcus* genus abundance; similarly the CXCL10 IP10 was associated with *R. ruminococcus* genus. Lastly, plasma IL‐12p70 was associated with *Phascolarctobacterium* genera as shown in Table 5.

## 4 Discussion

During the last decade, the microbiota structure has gained room in the research field for health promotion targets [1]. The gut microbiota is a relatively recent area of research that has been progressively linked with the pathophysiology of several diseases, showing its unexplored central role in homeostasis [58]. In this sense, microbiota reshaping seems to be a key adjuvant factor in interaction with long‐term health maintenance [2]. The present research highlights the effectiveness of a nutraceutical blend to induce weight loss mediated by the gut dysbiosis recovery reflected in the metabolic and inflammatory balance. The greatest find displayed here was related to the weight loss after only 90 days of supplementation without dietary restriction or a physical‐activity program, showing the anti‐obesity potential effect of the nutraceutical blend even in higher energy and carbohydrate intake scenarios. All the components in the formulation tested here were previously investigated as possible tools for obesity and metabolic disease treatments [8, 59–64]. However, the results presented here show the synergism of nutrients in the formulation powering up its beneficial effects. Thus, this clinical trial aims to elucidate the effects of a nutraceutical blend with prebiotics (β‐glucans, GOS, and FOS), essential minerals (zinc, magnesium, and selenium), associated or not with silymarin extract (an herbal medicine extract of *Silybum marianum* L. Gaertn., Asteraceae seeds), on metabolic and anthropometric measure improvement mediated by the gut microbiota reshape in 90 days of supplementation.

The formulation tested in this study was selected based on prior preclinical research that evaluated the effects of individual components and the complete nutraceutical blend on metabolic, endocrine, and microbiota modulation [12, 14, 65]. Building on this foundation, pilot trials were conducted to assess acceptance and the formulation's effects on metabolic and sleep outcomes in adults [20, 66]. Subsequently, translational studies examined microbiota modulation in both preclinical and clinical models [11]. Finally, extended supplementation periods were undertaken to investigate the blend's metabolic impact and its effects on the gut‐brain axis in greater depth [21, 22]. This study presents the results of 90 days of supplementation.

The anthropometric results show relevant modifications in body measures, especially central adiposity in a short time of supplementation without changes in lifestyle, highlighting the nutraceutical formulation's ability to promote positive metabolic changes. The increased neck circumference and WHtR are strongly related to overweight high cardiovascular risk [67]. The reduction of the anthropometric measures shows a body mass reduction based on visceral adipose tissue promoted by both nutraceutical supplements with a stronger effect related to the NSupple_*Silybum* group. The synergism of nutrients in the formulation might increase its beneficial effects by interacting with molecular mechanisms of action such as enhanced mitochondrial quantity and activity for energy production based on lipidic sources [68]. Previous research from our group on a preclinical model has proven the increased mitochondrial biogenesis under this nutraceutical formula test [13, 14]. The mitochondrial biogenesis might be a reasonable explanation for body measures reduction despite the increased carbohydrate or energy intake observed post‐supplementation. It is noteworthy that these changes occurred even in the absence of physical activity or dietary changes.

These anthropometric improvements come along with an improvement of several plasmatic markers post‐90 days of supplementation. Regarding the cardiovascular risk, the supplementation improved the atherogenic index, cholesterol profile, and liver injury markers, reflecting the cardiometabolic and hepatic protective effect of the supplement improving metabolic health, particularly in obesity‐related diseases [21]. These improvements suggest a positive shift in lipid metabolism and liver health, contributing to the overall management and prevention of metabolic disorders associated with obesity. Previous results suggest the interaction of gut microbiota on these parameters, revealing the appeal of prebiotics and nutraceuticals on dysbiosis and homeostasis recovery [11, 12]. Also, the stress‐related markers were improved, like cortisol level, by both supplements and the cortisol/C‐RP ratio in the NSupple group, as well as the TSH levels. The literature shows a relationship between cortisol and TSH levels with impaired sleep patterns and mood disorders besides its classical physiological roles [19, 69]. Even the cortisol/C‐RP ratio has been related to depressive symptoms [70]. Thus, the nutraceutical blends might act to improve metabolic and endocrine aspects contributing to homeostasis.

Another crucial factor for homeostasis is the inflammatory balance which is involved in several disease onset [71]. The results show that the NSupple_*Silybum* displayed a superior effect on the inflammatory molecules panel evaluated with a decrease in proinflammatory markers such as TNF‐α and CXCL10 IP‐10, as well as in anti‐inflammatory features like IL‐6/IL‐10 ratio and TNF‐α/IL‐10 ratio. These findings suggest a metainflammation recovery related to the supplementation using silymarin associated with the nutraceutical blend. It is well known that metabolic diseases like overweight, insulin resistance, and metabolic syndrome are closely associated with a persistent state of metainflammation that seems to be counteracted by the supplementation tested here [21, 72]. This effect might be related to the immune modulatory potential of the nutraceutical blend associated with the silymarin hepatoprotective effect, which acts synergistically to improve the immune response and resolve the overstimulated inflammatory process, bringing back the physiological balance.

The literature shows that sleep disorders can trigger increased inflammatory status; however, the underlying mechanisms are relatively unexplored [73]. Sleep influences the hypothalamus‐pituitary‐adrenal (HPA) axis and the sympathetic nervous system (SNS), shifting the basal gene expression profile toward increased proinflammatory status [74]. Proper nocturnal sleep is associated with a drop in sympathetic outflow, explaining the associations between sleep disturbance and increases in markers of inflammation [75]. The results highlight the volunteers' intermediate chronotype, which usually faces little difficulty adapting to regular working hours and rest schedules [76]. Even though our sample did not define a sleep disturbance population, the nutraceutical blends show improved sleep patterns after only 90 days of supplementation. These findings might be attributed to cortisol levels and inflammation markers recovery, such as IL‐10 and IL‐12p70, leading to better sleep quality. Sleep disruptions are often associated with an imbalance in these components, which can lead to mood disorders such as depression and anxiety [77]. The cellular mechanisms underlying these relationships reveal the complex interplay between sleep, inflammation, and mood regulation, with significant contributions from the gut microbiota [78].

Cortisol, the primary stress hormone, is a critical player in the relationship between sleep, mood, and psychological well‐being. Cortisol levels typically follow a circadian rhythm, with a peak in the early morning and a gradual decline throughout the day. Sleep disturbances can lead to dysregulation of this rhythm, often resulting in elevated evening cortisol levels [19]. Chronic elevation of cortisol due to poor sleep can have detrimental effects on mood. Elevated cortisol has been associated with hippocampal atrophy, impaired neurogenesis, and reduced synaptic plasticity, all of which are key factors in the development of depression and anxiety. Furthermore, high cortisol levels can promote the production of pro‐inflammatory cytokines, thereby linking the dysregulation of the HPA axis with increased inflammation and mood disturbances [79]. Our results reinforce the ability of psychological aspect improvement by this pathway along with the cytokines as cortisol modulation. The gut microbiota plays a significant role in modulating both the immune system and the HPA axis, influencing the levels of inflammatory cytokines and cortisol. The relationship between sleep, inflammatory cytokines, cortisol levels, and mood disorders is complex and multifaceted, involving intricate cellular mechanisms [15]. Inflammatory cytokines can disrupt sleep architecture and contribute to mood disorders, while dysregulated cortisol levels can exacerbate both sleep disturbances and mood imbalances [80]. The gut microbiota plays a crucial role in these processes, influencing both the immune system and the HPA axis. Therefore, the nutraceutical blend might be a tool for maintaining a healthy gut microbiota that may be key to improving sleep, reducing inflammation, and mitigating the risk of mood disorders.

Our data underscore the reshaping of gut microbiota following supplementation, with improvements observed in key bacterial taxa linked to obesity and metabolic disease prevention. The dominant phyla modulated in response to the intervention were Firmicutes and Bacteroidetes, which demonstrated a reduction in the F/B ratio, indicating a recovery from dysbiosis [3].

The increase in the Lentisphaerae phylum post‐supplementation marks a significant positive impact on the gut microbiota, particularly in relation to body weight reduction. Lentisphaerae, a lesser‐known phylum, has recently gained attention for its association with a leaner phenotype and metabolic benefits. Research indicates that the presence of Lentisphaerae is more prevalent in individuals with lower body weight, suggesting its role in promoting weight reduction by contributing to a healthier gut microbiota composition [81]. The reshaping of the microbiota by Lentisphaerae may help counteract dysbiosis commonly seen in obesity, fostering a microbial environment that supports metabolic balance [82]. Our findings align with these insights, showing a significant enrichment of the species *V. vadensis*, a key member of the Lentisphaerae phylum [83]. This species plays a pivotal role in fermenting carbohydrates to SCFAs, specifically acetic and butyric acid, which are known to influence energy regulation and reduce inflammation. The production of SCFAs supports gut barrier integrity, modulates immune responses, and indirectly influences weight management [83, 84]. Moreover, this microbial shift may enhance sleep quality by regulating the HPA axis, as demonstrated by the association between longer sleep duration and lower cortisol levels [85]. This connection between gut microbiota and HPA axis modulation underscores the gut‐brain axis’ role in sleep regulation, with potential implications for metabolic health improvements [19]. Overall, the increase in Lentisphaerae, particularly *V. vadensis*, emphasizes the positive reshaping of gut microbiota linked with body weight reduction, improved metabolic function, and enhanced sleep quality, showcasing the broader health benefits of microbiota‐targeted interventions.

Another noteworthy finding involves the *R. ruminococcus* genus, which was associated with both reduced body weight and lower inflammatory markers. Mechanistically, the primary carbohydrate degradation was predominantly carried out by Firmicutes, *with Ruminococcus* playing a key role in providing fermentation substrates that enhance acetate production [86]. This process supports the growth of butyrate‐producing bacteria that express the enzyme butyryl‐CoA: acetate CoA‐transferase [87]. The microbiota reshaping observed in our study likely triggered increased SCFA production, which in turn had beneficial effects on both stress regulation and sleep quality. The genus *Phascolarctobacterium* was also implicated in carbohydrate fermentation, specifically yielding propionate, a SCFA with anti‐lipogenic, cholesterol‐lowering, anti‐inflammatory, and anti‐carcinogenic properties [88]. Our findings confirm its anti‐inflammatory effects, as demonstrated by the association between *Phascolarctobacterium* and reduced plasma levels of IL‐12p70 cytokines. Elevated pro‐inflammatory cytokines, such as IL‐12p70, are known to contribute to the development of mood disorders, as they can cross the blood‐brain barrier and interact with brain regions responsible for mood regulation, including the hypothalamus and hippocampus [80].

The observed changes in gut microbiota, particularly the increase *in Faecalibacterium* and *Lactobacillus* genera, alongside the reduction in *Adlercreutzia* and *Sutterella*, reflect a significant shift towards a healthier microbial composition that likely contributes to improvements in obesity‐related metabolic dysfunction, sleep quality, and systemic inflammation promoted by the supplementation in the short term of 90 days. The increase in *F. prausnitzii*, a key butyrate‐producing species within the *Faecalibacterium* genus, is particularly notable due to its strong anti‐inflammatory properties [89]. Butyrate is essential for maintaining gut barrier integrity, modulating immune responses, and suppressing the production of pro‐inflammatory cytokines such as TNF‐α [26]. This reduction in inflammation is crucial, as chronic low‐grade inflammation is closely linked with both obesity and poor sleep quality [73], thus positioning *F. prausnitzii* as a pivotal player in mitigating these conditions.

Similarly, the rise in *Lactobacillus* genus supports improved metabolic outcomes and sleep patterns. Lactobacilli are known for their probiotic activity, enhancing the production of lactic acid and other SCFAs like acetate and butyrate, which influence gut health and immune regulation [90]. Moreover, *Lactobacillus* has been shown to reduce cortisol levels and modulate the HPA axis, potentially alleviating stress and promoting better sleep quality [91]. The reduction in cortisol and improvement in sleep duration likely result from this modulation of the HPA axis, which is known to be influenced by gut‐brain signaling [19]. The decrease in *Adlercreutzia* and *Sutterella* also points to beneficial shifts in gut microbiota. *Adlercreutzia* has been implicated in metabolic dysregulation, particularly in the context of obesity [92], while *Sutterella* has been associated with intestinal inflammation and gut permeability [93]. Reductions in these genera may thus reflect a healthier microbial environment, characterized by decreased inflammation and enhanced metabolic health.

At the species level, the increase in *Eubacterium biforme* and *B. eggerthii* further supports these improvements. *Eubacterium* species are known for their role in butyrate production, contributing to anti‐inflammatory effects and metabolic health [86]. *B. eggerthii*, a species commonly associated with gut health, plays a role in the fermentation of complex carbohydrates, producing beneficial SCFA [94]. Together, these species contribute to reduced gut inflammation and improved metabolic parameters, which are key in the context of obesity and related sleep disturbances [82]. Conversely, the reduction in species such as *C. eutactus, A. indistinctus, B. obeum, R. lactaris*, and *R. bromii* may reflect a shift away from microbial communities that are less beneficial in the context of metabolic health and inflammation. *C. eutactus*, while associated with SCFA production, has been linked with increased inflammation in certain contexts [95]. *A. indistinctus* and *B. obeum* have also been associated with dysbiosis and inflammatory profiles in obesity, suggesting that their reduction may be beneficial in promoting a more balanced and less inflammatory gut environment [96, 97]. *R. lactaris* and *R. bromii* are known starch degraders, and their reduction could signify a shift in carbohydrate metabolism, with potential implications for energy balance and fat storage [98, 99], which are crucial in the management of obesity.

The gut‐brain axis' role in mediating sleep and mood improvements observed in this study is compelling, though speculative. Emerging evidence supports that gut microbiota‐derived metabolites, such as SCFAs, modulate central nervous system functions via the *vagus* nerve and immune signaling pathways [15]. Future investigations could incorporate direct measurement of SCFAs, vagal nerve activity, or neuroinflammatory markers to confirm these mechanisms. Moreover, exploring the differential contributions of specific microbial taxa enriched in response to the nutraceutical blend, such as *F. prausnitzii* and *Lactobacillus spp*., might elucidate their role in regulating the hypothalamic‐pituitary‐adrenal axis and sleep quality [89]. Controlled experiments with neuroimaging or microbiota‐germ‐free models could further validate the hypothesized gut‐brain axis interactions. The observed improvements in sleep quality align with prior trials involving prebiotic supplementation, which reported enhanced SWS and reduced nocturnal awakenings [6]. However, the reduction in pro‐inflammatory cytokines, such as TNF‐α and CXCL10, suggests a stronger anti‐inflammatory effect than those studies, potentially attributable to the unique inclusion of silymarin. In contrast, earlier interventions using isolated prebiotics or polyphenols often showed limited effects on systemic inflammation in similar populations [61]. This distinction underscores the advantage of synergistic formulations.

Overall, these microbial changes highlight the complex interplay between gut microbiota composition and host metabolic health, inflammation, and sleep regulation [19, 85, 100]. The increase in beneficial genera such as *Faecalibacterium* and *Lactobacillus*, coupled with reductions in potentially harmful bacteria, suggests that microbiota modulation could be a promising therapeutic strategy for improving obesity, reducing systemic inflammation, and enhancing sleep quality. The specific species changes further underline the importance of SCFA production and inflammation regulation in this context, supporting the potential of targeted interventions that focus on reshaping the gut microbiota for optimal metabolic and sleep outcomes.

Sleep is a critical component of overall health, intricately linked to various physiological processes, including the regulation of inflammatory cytokines and cortisol levels. Thus, the results presented here allow us to infer that the nutraceutical blend tested was able to improve diverse health aspects, from body measures to psychological aspects, through the microbiota composition reshape. While the absence of a placebo group in this clinical trial may seem unconventional, in gut microbiota research, common placebos such as starch, resistant starch, cellulose, sugar, and maltodextrin can interact with the gut microbiota, potentially biasing the results by altering the microbiota composition, producing metabolites utilized by intestinal cells, and entering the bloodstream [101, 102, 103, 104, 105, 106, 107, 108]. Given the lack of an ideal placebo that does not interact with gut bacteria, we opted to forgo a placebo and instead used a self‐controlled design, comparing participants' data before and after supplementation in paired analyses. Nonetheless, it is crucial to acknowledge the study's inherent limitations; these include the small sample size, the unequal gender distribution, the use of subsampling for microbiota analysis, and the lack of in‐depth mechanistic analysis.

## Conflicts of Interest

This study received funding from EFEOM Nutrition S.A. (01/04‐21). The funder had the following involvement with the study: financial support for experimental protocol development and financial resources for the analysis performed. All authors declare no other competing interests.

## Authors Contributions

A.F.M.P. and J.P.O. supervised the study and designed experiments. J.V.F., M.T.H., A.F.G., and J.A.F. carried out experiments. A.F.M.P., J.A.F., and A.B.S. prepared the manuscript. J.A.F., R.C.M., J.V.F., E.H.R.O., and L.A.M.F. assisted in the data analysis. J.P.O., E.A.S., and V.N.F. viewed the manuscript. V.N.F. is the main funder of this study. D.P.D., B.F.R.B.S., and J.A.T. provided discussion of findings, and statistical analysis. A.F.M.P. provided the conception and design of the study and was an advisor and supervisor throughout the project.

## Supporting information



Supporting Information

## Data Availability

The datasets generated and/or analyzed during the current study are available in the GenBank repository, Bioproject PRJNA941000. Link: https://dataview.ncbi.nlm.nih.gov/object/PRJNA941000 (Release date: 2024‐09‐30). This link is exclusive for the reviewers: https://dataview.ncbi.nlm.nih.gov/object/PRJNA941000?reviewer = lfevhd0p2jn9358imrei528946
