# Supplementary material for: Nutraceutical Blends Promote Weight Loss, Inflammation Reduction, and Better Sleep: The Role of Faecalibacterium prausnitzii in Overweight Adults–A Double‐Blind Trial
Source: Mol Nutr Food Res. 2025 Feb 21;69(17):e202400806. doi: 10.1002/mnfr.202400806 (PMC12410511; doi:10.1002/mnfr.202400806)
Supplement: Supplementary file 1 — Supporting Information [file MNFR-69-e202400806-s001.docx]

**Supplementary data**

**Table 1S**. Anthropometric characterization, food intake, and serum parameters in the study population before and after the supplementation.

| Anthropometrics | | | | | | |
| --- | --- | --- | --- | --- | --- | --- |
|  | **NSupple** | |  | **NSupple_*Silybum*** | |  |
|  | **T0** | **T90** |  | **T0** | **T90** |  |
|  | Mean±SD | Mean±SD | *p* | Mean±SD | Mean±SD | *p* |
| WHR | 0.86±0.01 | 0.86±0.01 | *-* | 0.87±0.01 | 0.87±0.01 | *-* |
| Serum parameters | | | | | | |
|  | **NSupple** | |  | **NSupple_*Silybum*** | |  |
|  | **T0** | **T90** |  | **T0** | **T90** |  |
|  | Mean±SD | Mean±SD | *p* | Mean±SD | Mean±SD | *p* |
| Non-HDL-c (mg/dL) | 164.6±6.31 | 173.8±8.25 | *-* | 154.4±6.84 | 148.2±5.65 | *-* |
| Gamma-GT (U/L) | 19.43±1.93 | 21.38±2.19 | *-* | 21.59±2.21 | 22.56±2.27 | *-* |
| HDL-c (mg/dL) | 54.17±2.45 | 54.92±2.53 | *-* | 50.63±2.17 | 51.56±2.14 | *-* |
| HOMA-IR | 2.48±0.22 | 2.188±0.16 | *-* | 2.59±0.26 | 2.76±0.33 | *-* |
| IgA (mg/dL) | 244.3±21.04 | 227.8±16.37 | *-* | 208.6±12.49 | 214.7±13.37 | *-* |
| IgG (mg/dL) | 1157±44.38 | 1135±36.56 | *-* | 1090±37.13 | 1086±33.23 | *-* |
| Insulin (mU/L) | 12.03±1.09 | 10.27±0.73 | *-* | 12.26±1.13 | 9.11±0.82 | *-* |
| C-Reactive protein (mg/dL) | 0.20±0.02 | 0.16±0.02 | *-* | 0.19±0.02 | 0.16±0.02 | *-* |
| Thyroxine (ng/dL) | 0.92±0.02 | 0.93±0.03 | *-* | 0.97±0.02 | 0.99±0.03 | *-* |
| AST (U/L) | 19.89±1.03 | 18.26±0.76 | *-* | 19.69±1.18 | 18.26±0.84 | *-* |
| Triglycerides (mg/dL) | 119.9±6.51 | 122.8±8.38 | *-* | 140.2±10.19 | 133.4±9.56 | *-* |
| VLDL-c (mg/dL) | 23.37±0.94 | 23.84±1.22 | *-* | 25.59±1.35 | 23.97±1.07 | *-* |

BMI: body mass index; WHR: waist-to-hip ratio; HDL-c: high-density lipoproteins cholesterol; AST: aspartate aminotransferase; VLDL-c: very-low-density lipoprotein cholesterol.

**Table 2S.** Cytokines and chemokines expression in overweight volunteers after 90 days of supplementation.

| Group | NSupple | |  | NSupple_*Silybum* | |  |
| --- | --- | --- | --- | --- | --- | --- |
|  | **T0** | **T90** | *p* | **T0** | **T90** | *p* |
| (pg/mL) | Mean±SD | Mean±SD | - | Mean±SD | Mean±SD | - |
| IL-1β | 1.31±3.25 | 0.8144±1.31 | - | 1.26±1.75 | 0.99±1.53 | - |
| IL-6 | 1.03±0.83 | 1.005±0.59 | - | 0.80±0.67 | 0.68±0.61 | - |
| IL-8 | 1.37±4.86 | 0.7849±1.46 | - | 0.52±0.93 | 0.26±0.65 | - |
| IL-10 | 3.23±2.36 | 2.938±1.05 | - | 2.862±1.69 | 2.66±0.98 | - |
| CCL5/RANTES | 42.52±23.16 | 41.41±24.05 | - | 33.51±26.20 | 31.71±29.11 | - |

**Table 3S.** Sleep quality (MSQ-BR) and mood characterization (BRUMS) in the study population before and after the supplementation.

| NSupple | | | | | | NSupple_*Silybum* | | | | |
| --- | --- | --- | --- | --- | --- | --- | --- | --- | --- | --- |
|  | **T0** | | **T90** | | *p* | **T0** | | **T90** | | *p* |
|  | Mean±SD | CI 95% | Mean±SD | CI 95% |  | Mean±SD | CI 95% | Mean±SD | CI 95% |  |
| Mini-Sleep Questionnaire (MSQ-BR) | | | | | | | | | | |
| MSQ-BR Score | 32.49±1.49 | 29.49 - 35.49 | 31.11±1.64 | 27.77 - 34.45 | *-* | 27.63±1.22 | 25.18 - 30.08 | 26.73±1.21 | 24.27 - 29.18 | *-* |
| Brunel Mood Scale (BRUMS) | | | | | | | | | | |
| BRUMS total score | 20.91±1.93 | 17.02 - 24.80 | 22.69±2.05 | 18.52 - 26.85 | *-* | 24.65±2.18 | 20.25 - 29.05 | 25.9±2.33 | 21.18 - 30.62 | *-* |
| Tension | 4.26±0.28 | 3.70 - 4.81 | 4.06±0.27 | 3.51 - 4.60 | *-* | 4.41±0.29 | 3.83 - 5.00 | 4.43±0.26 | 3.91 - 4.94 | *-* |
| Depression | 2.67±0.40 | 1.87 - 3.48 | 3.01±0.43 | 2.13 - 3.87 | *-* | 3.17±0.48 | 2.21 - 4.13 | 3.65±0.51 | 2.63 - 4.67 | *-* |
| Anger | 2.23±0.33 | 1.56 - 2.91 | 2.69±0.31 | 2.06 - 3.31 | *-* | 3.11±0.45 | 2.21 - 4.01 | 3.63±0.49 | 2.64 - 4.61 | *-* |
| Vigor | 4.61±0.36 | 3.88 - 5.33 | 4.80±0.40 | 3.99 - 5.61 | *-* | 5.78±0.35 | 5.07 - 6.50 | 5.50±0.40 | 4.70 - 6.30 | *-* |
| Fatigue | 3.14±0.27 | 2.60 - 3.68 | 3.51±0.34 | 2.83 - 4.20 | *-* | 3.72±0.28 | 3.16 - 4.28 | 3.85±0.31 | 3.23 - 4.47 | *-* |
| Confusion | 4.00±0.30 | 3.40 - 4.60 | 4.63±0.31 | 4.01 - 5.25 | *-* | 4.46±0.34 | 3.77 - 5.14 | 4.85±0.38 | 4.08 - 5.62 | *-* |


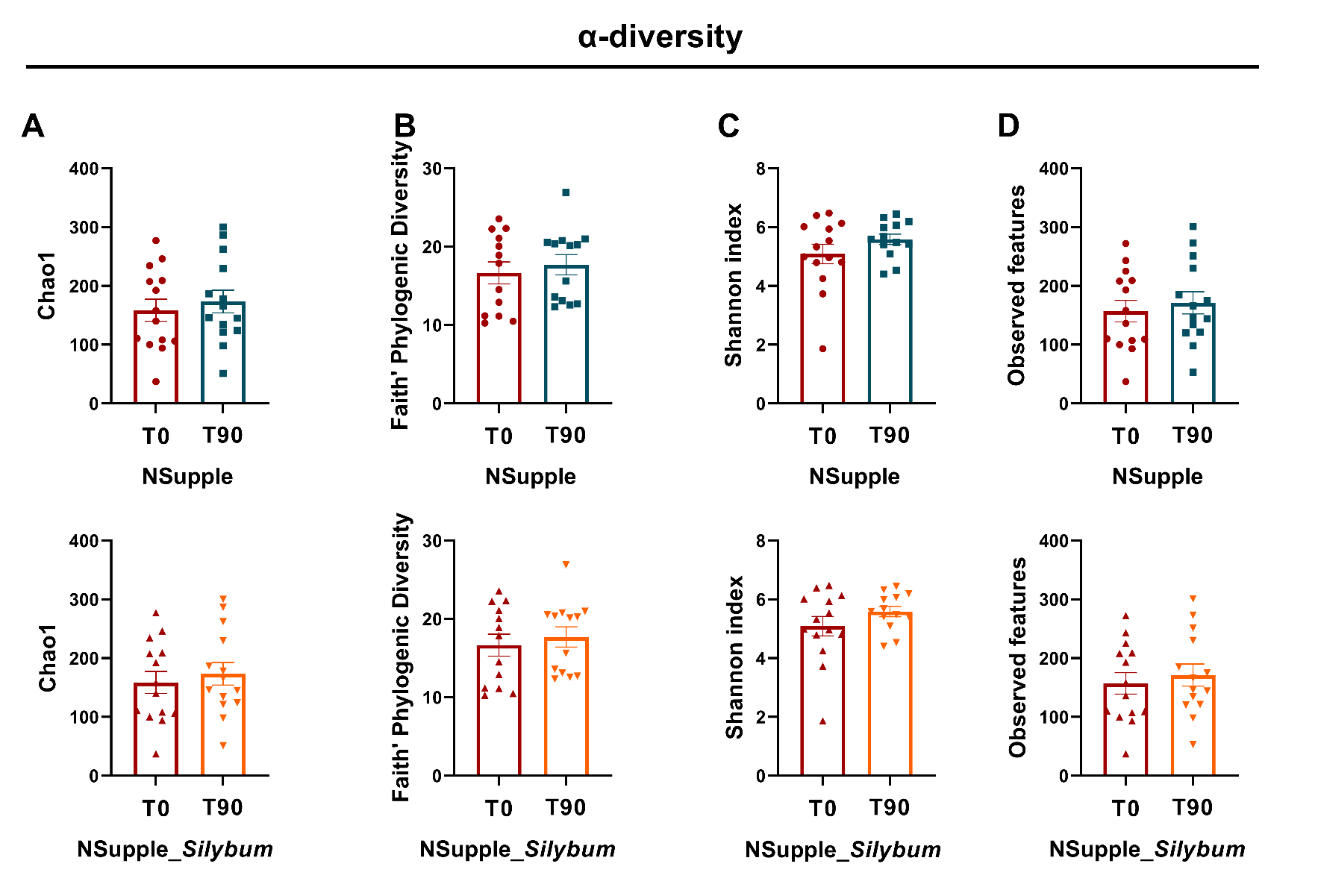


**Figure 1S.** Alpha (α) diversity indexes evaluated before and after supplementation in NSupple and NSupple_*Silybum* groups: **[A]** Chao1, **[B]** Faith’ Phylogenic Diversity, **[C]** Shannon index, **[D]** Observed features. Values are expressed as the percent of relative abundance (mean ± standard deviation).
